# Cellular radiosensitivity: do separate predictive parameters apply for fibroblasts and for human tumour cells?

**DOI:** 10.1038/sj.bjc.6601572

**Published:** 2004-01-20

**Authors:** L Bohm

**Affiliations:** 1 Department of Pharmacology, University of Stellenbosch, Tygerberg 7505, South Africa

Sir,

A paper by El-Awady *et al*, 2003 brings to the fore new evidence for correlations between radiosensitivity and the initial DNA damage in the form of double-strand breaks (dsb). The conclusion is at variance with a previous paper by the same group where the nonrepaired DNA dsb were identified as indicators of radiosensitivity (Dikomey *et al*, 1998). Both studies use constant field gel electrophoresis (CFGE) and a modification called graded field gel electrophoresis (GFGE) that detects mobile low molecular weight DNA fragments generated by large single doses of irradiation in the range of 20–100 Gy (Dahm-Daphi and Dikomey, 1995). This dose range is far above the dose fractions used in tumour treatment, but is essential to generate a detectable level of DNA fragments. Using low doses of irradiation and alkaline unwinding to remove all damaged DNA, the fraction of intact double-stranded DNA that declines with dose was found to be essentially the same between the nine cell lines, a result confirmed by the CFGE technique after high-dose exposures (Dikomey *et al*, 1998). It is therefore somewhat puzzling if not paradoxical that the same methodology of CFGE should show initial dsb to correlate with radiosensitivity in one set of cell lines (El-Awady *et al*, 2003) and nonrepaired dsb in another (Dikomey *et al*, 1998).

However, disagreement between studies has existed for a good 15 years now. The new CFGE measurements (El-Awady *et al*, 2003) showing a correlation between initial damage and radiosensitivity in human tumour cell lines are in agreement with three earlier investigations (McMillan *et al*, 1990; Ruiz de Almodovar *et al*, 1994; Whitaker *et al*, 1995; McMillan *et al*, 2001). A total of 10 other studies fully referenced in El-Awady *et al* have remained less conclusive. Reference to the important data of Radford (1985), (1986), Prise *et al* (1987), Prise *et al*, 1989 and very recent work of Roos *et al* (2000) based on unwinding would have shown that a strong correlation between initial damage and radiosensitivity had previously been demonstrated in fibroblasts. Another point of interest is that five of the 11 cell lines used by Roos *et al* were also part of the CFGE study showing a correlation between unrepaired dsb and radiosensitivity (Dikomey *et al*, 1998, 2000). It now appears that this correlation is not valid for human tumour cells (El-Awady *et al*, 2003). A relationship between repair fidelity and radiosensitivity mainly based on a plasmid transfection assay and recovery of restriction cuts has also been reported (Powell and McMillan, 1994). In recent CFGE measurements on six human prostate tumour cell lines (some derived from primary tumour epithelium), we report a significant correlation between the 2 h repair capacity but not with the initial DNA damage (Serafin *et al*, 2003). From the above, one cannot escape the conclusion that a consensus view of which single parameter would predict radiosensitivity has not yet emerged.

Events measured after 20–24 h of repair when compared and correlated with cell survival measured after 7–10 days have to deal with the problem that nonrepaired and repaired dsb in fact are early consequences of the irradiation insult. At 20–24 h postirradiation, the repaired DNA would be found in the total envelope of undamaged DNA, but may be functionally compromised by misrepair and say little about the performance of the restituted DNA strands in subsequent rounds of cell division. It seems likely therefore that determination of nonrepaired dsb underestimates the full biological damage that manifests itself only after 7–10 cell divisions when mitotic integrity can be recognised and quantitated from the surviving clonogens. The induced dsb (or the induced DNA damage), on the other hand, measured at zero time and in the absence of any repair conceivably represents initial damage that would depend on cell type and dose alone, hence not incorporating any time-dependent variables affecting the comparison with cell survival. That broad variations found in the amount of dsb remaining after 24 h of repair in tumour cells may arise from apoptosis, and cell cycle progression as suggested (El-Awady *et al*, 2003) would underline the principal uncertainties associated with the 24 h repair marker and early damage responses in general. The p53 status of the tumour lines used by El-Awady *et al* (2003) was not identified further, but inspection shows that the cell lines are heterogeneous some being wild type, some being mutant and some being dysfunctional. It is clear therefore that the check point and S-phase arguments would apply differently to the tumour cell lines analysed by El-Awady *et al* (2003).

A methodological distinction worth noting is that the fluorescent analysis of DNA unwinding (FADU) method (Birnboim and Jevcak, 1981 and Ogiu *et al*, 1992) operates in the clinical dose range of 1–10 Gy and emerges as much more sensitive than CFGE. At low dose and especially at the clinically relevant survival level of 2 Gy, the *α*/*β* model would predict survival to arise mainly from single event inactivation (*α*-damage) irrespective of the radiosensitivity (Steel and Peacock, 1989). In the GFGE method, the slope of the DNA release curve varies by a factor of 2 between nine cell lines (see Table 2 in El-Awady *et al*, 2003), whereas slopes in the damage induction curve between 11 cell lines analysed by the FADU method operating at low dose vary by a factor of 6.3 (Roos *et al*, 2000), thus achieving a much better separation. In the same study, we also show that repair competent cell lines differing widely in radiosensitivity repair 94–98% of their damaged DNA within 12 h with no apparent correlation between residual damage and radiosensitivity. Thus it could be an inherent phenomenon of the cell lines that the less sensitive GFGE method should identify initial damage as an indicator of radiosensitivity, as suggested (El-Awady *et al*, 2003). It could also be a reflection of the superior ranking of initial DNA damage as a determinant for cell survival. More measurments using the fluorescent unwinding technique could help to clarify.

Recently, it has been suggested that identical lesions may be recognised differently by different cell lines (Olive, 1998). It is also accepted that cell signalling and apoptotic death can be invoked by damage other than dsb (McMillan *et al*, 2001). A case has been made to show that the initial damage translates into a variety of early damage responses that are dose dependent and vary widely in magnitude between cell types (Akudugu and Bohm, 2001). Attempts to reconstruct cell survival from early response parameters such as micronuclei, apoptosis and abnormal nuclear morphology have highlighted these differences and led to the postulate of unknown events (Abend *et al*, 2000; Akudugu *et al*, 2000), showing that it is not possible to reconstruct radiosensitivity from any one of these parameters (Akudugu and Bohm, 2001; Akudugu *et al*, 2002).

In conclusion, the new evidence from high-dose experiments and CFGE that the initial DNA damage correlates with radiosensitivity in tumour but not in fibroblast cell lines strictly holds only for the CFGE method. The FADU unwinding method operating at low dose (and other methods) also generate such a relationship in fibroblast cell lines. The observed correlations thus would depend upon methodology and other unknown factors. The fact that DNA dsb are a nonuniform group of lesions (Ward, 1988) and differ widely in terms of lethality (Prise *et al*, 1989) suggests that the dose range may be important. A pragmatic way forward could be to examine the validity of the new correlations for predictive purposes in the clinic.
